# Recent advances in understanding basophil functions *in vivo*

**DOI:** 10.12688/f1000research.11697.1

**Published:** 2017-08-15

**Authors:** David Voehringer

**Affiliations:** 1 Department of Infection Biology, University Hospital Erlangen, Erlangen, Germany

**Keywords:** basophil, transcription factors, antigen-presentation, inflammatory response, allergy

## Abstract

Basophils are mainly known as pro-inflammatory effector cells associated with allergy and helminth infections. Although they were identified over 130 years ago, their *in vivo* functions are still poorly understood. New insights into basophil development and function have been gained by the development of various transgenic mouse lines and staining techniques to detect and purify these cells from different organs. Several studies over the past few years have identified unexpected functions for basophils, including immunomodulatory properties and interactions with other immune cells. Here, I summarize and discuss the main findings.

## Introduction

Basophils belong to the group of granulocytes which constitute rather short-lived effector cells of the innate immune system. They usually represent less than 1% of all leukocytes in the peripheral blood, but they have potent effector functions. In contrast to many other cell types of the immune system, it is poorly understood how basophils develop and execute their effector functions. Not too long ago, it was even questioned whether mice contain a bona fide basophil population mainly because mouse basophils contain fewer granules as compared to human basophils ^1^. After establishing staining protocols and genetically engineered mouse strains basophils could be identified, isolated, and functionally characterized in various settings of immune responses. Basophils and mast cells share the expression of the high-affinity receptor for IgE (FcεRI), histamine, and a few other effector molecules, yet basophils do not represent a subset or precursor population of mast cells; rather, they constitute a distinct cell lineage with a very different gene expression profile ^2^. Basophils can be efficiently depleted *in vivo* with the monoclonal antibody MAR-1 directed against FcεRI or Ba103 which binds to another activating receptor named CD200R3. However, since the recognized antigens of both antibodies are not exclusively expressed on basophils, this approach can cause bystander effects that interfere with clear interpretations of such depletion experiments. Transgenic mice expressing the Cre recombinase, GFP, or human diphtheria-toxin receptor under control of regulatory elements of the *Mcpt8* gene have been developed by several groups over the past few years (reviewed in 3). *Mcpt8* encodes the mouse mast cell protease 8 (mMCP-8), a serine protease which is highly expressed in basophils but not mast cells ^4^. The genetically modified mouse strains facilitate the functional characterization of basophils *in vivo*. However, it is important to keep in mind that mouse and human basophils differ in many respects, and it remains to be determined to what extent findings in mouse models can be translated to the human immune system. In the following paragraphs, I summarize some major new findings regarding the *in vivo* functions of basophils published during the past few years.

## Basophil development and critical transcription factors

Interleukin (IL)-3 is the most potent cytokine to promote basophil proliferation, but high concentrations of thymic stromal lymphopoietin (TSLP) can have similar effects ^5^. IL-3 and TSLP-elicited basophils differ in terms of their gene expression profiles and may resemble different states of activation rather than representing stable subpopulations of basophils. The receptors for IL-3 and TSLP are linked to the STAT5 signaling pathway, and it was recently shown that STAT5 binds to regulatory elements of Gata2, which is another critical transcription factor for basophil development ^6^. In addition, it was found that IRF8 can also promote Gata2 expression in precursor cells and thereby drive basophil differentiation ^7^. Other transcription factors including Gata1, P1-Runx1, c/EBPα, and MITF also play an important role in basophil development and maintenance (reviewed in 8).

## Relevance of antigen-presentation by basophils

Basophils were found to display low levels of major histocompatibility complex class II (MHC-II) on the cell surface, and antibody-mediated depletion of basophils resulted in poor Th2 polarization ^9– 11^. However, genetically basophil-depleted *Mcpt8Cre* mice showed normal expansion of Th2 cells during primary infection with the helminths *Nippostrongylus brasiliensis* ^12^, *Heligmosomoides polygyrus* ^13^, and *Schistosoma mansoni* ^14^. Ovalbumin (OVA)-alum immunized and challenged *Mcpt8Cre* mice also showed an unimpaired Th2 response and eosinophilia in the lung ^12^. Furthermore, papain+OVA-induced T-cell proliferation and Th2 polarization in draining lymph nodes was normal in *Mcpt8Cre* mice while genetically dendritic cell (DC)-depleted mice showed a severely impaired response ^12^. With another genetically basophil-depleted mouse model ( *Basoph8* x *Rosa-DTa* mice), it was shown that footpad immunization with *S. mansoni* eggs results in normal Th2 priming in the absence of basophils ^15^. In contrast, diphtheria toxin (DT)-mediated depletion of DCs causes impaired Th2 priming upon *S. mansoni* egg immunization and diminished Th2 cell accumulation in the liver of *S. mansoni*-infected mice ^16^. It was further shown that DCs are required and sufficient for the Th2 response to house dust mite antigens ^17^. This study also demonstrated that a subset of monocyte-derived DCs express FcεRI. These cells will therefore also be depleted with the anti-FcεRI antibody MAR-1 frequently used to deplete basophils *in vivo*.

Basophils lack the machinery for antigen uptake and processing, although they can contain MHC-II molecules on the cell surface which may be loaded with exogenous peptides and are then capable of stimulating T cells ^9, 18, 19^. Two other studies provide evidence that basophils and DCs cooperate to promote Th2 polarization. It was shown that subcutaneous papain injection induced reactive oxygen species that indirectly activated DCs to promote basophil recruitment into lymph nodes and subsequent Th2 polarization ^20^. Another study showed that TSLP-elicited dermal DCs express OX40L to induce IL-3 secretion from T cells leading to the recruitment of basophils which then promote Th2 polarization ^21^. The Th2-promoting activity of basophils in both studies might be explained by basophil-derived IL-4 rather than direct antigen recognition on basophils.

Recent evidence shows that basophils can in fact acquire MHC-II from DCs by the uptake of plasma membrane patches, a process termed trogocytosis ^19^. Clearly, further studies are needed to determine whether MHC-II trogocytosis by basophils has functional consequences for T-cell activation or memory formation or other processes that are regulated by antigen recognition.

## Basophil functions in type 2 immune responses

### Lung inflammation

In a mouse model of allergic lung inflammation induced by the administration of the cysteine protease papain, it was found that basophil-derived IL-4 promotes the secretion of IL-5, IL-9, and IL-13 from type 2 innate lymphoid cells (ILC2s) in the lung and thereby induces lung eosinophilia ^22^. Another type 2 immunity-inducing property of basophils was observed in a commonly used model for allergic lung inflammation which is based on alum adjuvant-mediated priming of the Th2 response. It was recently reported that alum enhanced the ability of basophils to induce Th2 polarization by the release of TSLP and IL-25 but independently of IL-4 secretion ^23^. However, we and others observed no impairment in lung Th2 responses with *N. brasiliensis* infection or OVA/alum immunization in genetically basophil-depleted mice ^12, 15^.

### Local inflammatory responses in the skin

Similar to reports from papain-induced lung inflammation, it was reported that basophil-derived IL-4 induces ILC2 accumulation and proliferation in the skin after topical application of MC903, a vitamin D analog ^24^. MC903 elicits high levels of TSLP expression in the skin and causes pathology reminiscent of skin lesions of atopic dermatitis patients. Anti-TSLP antibodies can block the accumulation of basophils in the skin ^25^ but also inhibit peripheral TSLP-induced basophilia during *Trichinella spiralis* infection ^26^. Experiments with mixed bone marrow chimeras revealed that basophil accumulation in the MC903-treated ear does not require direct recognition of TSLP by basophils ^5^. Basophils were further found to cooperate with fibroblasts to promote the recruitment of eosinophils in a murine model of irritant contact dermatitis ^27^. Related to this, it was reported that basophils regulate the entry of eosinophils into the skin by the induction of VCAM-1 expression on endothelial cells ^28^.

Local activation of basophils in the ear skin via FcεRI causes an ear swelling response termed “chronic allergic inflammation” or IgE-CAI, which peaks at day 2–3 and resolves by day 6. This IgE-CAI response is strictly dependent on basophils ^29^. Using this model, Egawa *et al*. reported that the release of IL-4 from basophils promotes the differentiation of alternatively activated or “M2” macrophages which have anti-inflammatory and tissue repair properties, arguing for a role of basophils in the resolution of inflammation ^30^. Others observed that α(1,3) fucosyltransferases IV and VII are essential for the initial recruitment of basophils in the IgE-CAI model ^31^. Basophils express various proteases including mMCP-8 and mMCP-11. By analyzing mMCP-11-deficient mice, Iki *et al*. showed that mMCP-11 promotes the ear swelling response in IgE-CAI ^32^. However, basophils can also have anti-inflammatory properties, as shown in a model of skin contact hypersensitivity where UVB irradiation reduced the hapten-induced ear swelling response. In this model, it could be demonstrated that basophil-restricted expression of amphiregulin, a cytokine with tissue repair activities, was required for the suppressive effect of UVB irradiation ^33^. Increased numbers of basophils were also found in skin biopsies from patients with various skin disorders, indicating that these cells also regulate inflammatory responses in human skin ^34^.

### Protection against parasites in the skin and intestine

The presence of basophils in the skin indicates that they could be involved in protective immunity against helminths that enter their hosts via the skin or against ectoparasites like ticks. Using basophil-depleted mice, Wada *et al*. showed that basophils impair tick feeding upon secondary engorgement ^35^. Furthermore, basophils promote the trapping of larvae in the skin upon secondary *N. brasiliensis* infection ^36^. In addition, basophils accumulate in the small intestine and induce rapid expansion of Th2 cells and protection against *N. brasiliensis* or *H. polygyrus* during secondary infection by IgE-induced secretion of IL-4/IL-13 ^13^. Basophils further promote a Th2 response in a TSLP-dependent manner during *T. spiralis* and *Trichuris muris* infections ^25, 26^. In contrast, basophils play only a minor role in the control of *Strongyloides ratti* infection ^37^. Anti-CD200R3-mediated depletion of basophils resulted in smaller granulomas around *S. mansoni* eggs in the liver ^38^, while this effect was not observed in genetically basophil-depleted *Mcpt8Cre* mice ^14^. This difference might be explained by secondary effects caused by the injected antibody, including mast cell activation and Fc receptor-mediated modulation of phagocytes.

## Basophils in food allergy and eosinophilic esophagitis

An interesting role for basophils during skin sensitization followed by oral challenge to elicit a food allergic response has been described. Mice developed a severe food allergic response when chicken OVA was first applied in combination with MC903 to the ear skin and later given intragastrically by oral gavage ^39^. The authors further showed that basophils and TSLP are required for this effect. In a subsequent study, it was further revealed that basophil-derived IL-4 promoted the IgE-mediated food allergic response and eosinophils were dispensable for this effect ^40^. A role for basophils, TSLP, and IL-33 to elicit an anaphylactic response to oral antigens was also shown in another model where OVA was applied to skin pretreated with 4% SDS instead of MC903 ^41^.

The impaired skin barrier function in atopic dermatitis may facilitate allergic sensitization that can lead to eosinophilic esophagitis (EoE). Pathology reminiscent of EoE can be elicited by intranasal OVA challenge of MC903+OVA skin-sensitized mice ^42^. Using this model, Noti *et al*. showed that IgE is dispensable for EoE while basophils and TSLP are required ^42^. Furthermore, IL-33 was identified as a critical cytokine to promote the accumulation of basophils in the esophagus and the recruitment of eosinophils ^43^.

## Basophils in other inflammatory settings, allograft transplantation, and tumor control

Intravenous immunoglobulin (IVIG) therapy, the intravenous administration of high doses of purified IgG, is used as a therapeutic approach to treat autoantibody-mediated inflammation in various clinical settings. The anti-inflammatory mechanism of IVIG therapy is incompletely understood. Using a mouse model of serum-induced arthritis, researchers showed that IVIG elicits IL-33 secretion, which in turn promotes IL-4 release from basophils. Basophil-derived IL-4 then upregulates the inhibitory Fc receptor FcγRIIB on macrophages and thereby ameliorates pathology ^44^. However, others found no role for basophils in IVIG-induced suppression of serum-induced arthritis ^45^. The apparent discrepancies between these observations remain to be resolved.

Systemic lupus erythematosus (SLE) is another autoantibody-mediated disease. SLE patients were found to have higher serum levels of IgE and activated basophils ^46^. Another study showed that basophils from SLE patients promoted Th17 differentiation *in vitro*, probably by the secretion of IL-6 ^47^. Depletion of basophils from SLE-prone MRL-lpr/lpr mice resulted in ameliorated pathology and an extended lifespan, while adoptive transfer of basophils had the opposite effect ^47^. Further evidence for an important role of basophil-derived IL-6 in Th17 differentiation is based on a cholera toxin-induced lung inflammation model. Here it was shown that Th17-associated lung inflammation was reduced in the absence of basophils and could be restored by the transfer of wild-type but not IL-6-deficient basophils ^48^.

Basophils were also found to regulate the rejection of allogeneic transplants. In a mouse model of pancreatic islet allotransplantation, it was reported that the depletion of basophils results in improved graft survival ^49^. Furthermore, basophil-derived IL-4 was found to act on myofibroblasts and promote fibrosis in a cardiac allotransplantation model ^50^. *In vitro* studies with human basophils further revealed their potential to inhibit TLR4-induced monocyte activation ^51^ and to induce the differentiation of alternatively activated macrophages ^52^.

Recently, basophils were further found to modulate immune responses against solid tumors. One study described the recruitment of basophils into tumor-draining lymph nodes in correlation with a Th2-biased immune response and poor survival of pancreatic cancer patients ^53^. In contrast, basophils were also shown to promote tumor rejection by recruiting CD8+ T cells.

## Future perspective

Our current understanding of basophil development and effector functions has improved considerably over the past few years. We realize now that basophils not only function as pro-inflammatory cells during allergic responses and helminth infections but also modulate the immune system in many ways. Most of our knowledge is still based on mouse models. It will be important to translate these findings to the human immune system in order to develop new therapeutic approaches for the treatment of inflammatory diseases where basophils may play an important role.

## Abbreviations

DC, dendritic cell; EoE, eosinophilic esophagitis; FcεRI, high-affinity IgE receptor; IL, interleukin; ILC2, type 2 innate lymphoid cells; IVIG, intravenous immunoglobulin; MHC-II, major histocompatibility complex class II; mMCP-8, mouse mast cell protease 8; OVA, ovalbumin; SLE, systemic lupus erythematosus; TSLP, thymic stromal lymphopoietin.

## References

[1] Lee JJ McGarry MP: When is a mouse basophil not a basophil? *Blood.* 2007;109(3):859–61. 10.1182/blood-2006-06-027490 17003367 PMC1785136

[2] Dwyer DF Barrett NA Austen KF: Expression profiling of constitutive mast cells reveals a unique identity within the immune system. *Nat Immunol.* 2016;17(7):878–87. 10.1038/ni.3445 27135604 PMC5045264

[3] Voehringer D: Protective and pathological roles of mast cells and basophils. *Nat Rev Immunol.* 2013;13(5):362–75. 10.1038/nri3427 23558889

[4] Lützelschwab C Huang MR Kullberg MC: Characterization of mouse mast cell protease-8, the first member of a novel subfamily of mouse mast cell serine proteases, distinct from both the classical chymases and tryptases. *Eur J Immunol.* 1998;28(3):1022–33. 9541598 10.1002/(SICI)1521-4141(199803)28:03<1022::AID-IMMU1022>3.0.CO;2-1

[5] Schwartz C Eberle JU Hoyler T: Opposing functions of thymic stromal lymphopoietin-responsive basophils and dendritic cells in a mouse model of atopic dermatitis. *J Allergy Clin Immunol.* 2016;138(5):1443–1446.e8. 10.1016/j.jaci.2016.04.031 27372565

[6] Li Y Qi X Liu B: The STAT5-GATA2 pathway is critical in basophil and mast cell differentiation and maintenance. *J Immunol.* 2015;194(9):4328–38. 10.4049/jimmunol.1500018 25801432 PMC4405376

[7] Sasaki H Kurotaki D Osato N: Transcription factor IRF8 plays a critical role in the development of murine basophils and mast cells. *Blood.* 2015;125(2):358–69. 10.1182/blood-2014-02-557983 25398936 PMC4287642

[8] Huang H Li Y Liu B: Transcriptional regulation of mast cell and basophil lineage commitment. *Semin Immunopathol.* 2016;38(5):539–48. 10.1007/s00281-016-0562-4 27126100 PMC5010465

[9] Perrigoue JG Saenz SA Siracusa MC: MHC class II-dependent basophil-CD4 ^+^ T cell interactions promote T _H_2 cytokine-dependent immunity. *Nat Immunol.* 2009;10(7):697–705. 10.1038/ni.1740 19465906 PMC2711559

[10] Sokol CL Chu NQ Yu S: Basophils function as antigen-presenting cells for an allergen-induced T helper type 2 response. *Nat Immunol.* 2009;10(7):713–20. 10.1038/ni.1738 19465907 PMC3252751

[11] Yoshimoto T Yasuda K Tanaka H: Basophils contribute to T _H_2-IgE responses *in vivo* via IL-4 production and presentation of peptide-MHC class II complexes to CD4 ^+^ T cells. *Nat Immunol.* 2009;10(7):706–12. 10.1038/ni.1737 19465908

[12] Ohnmacht C Schwartz C Panzer M: Basophils orchestrate chronic allergic dermatitis and protective immunity against helminths. *Immunity.* 2010;33(3):364–74. 10.1016/j.immuni.2010.08.011 20817571

[13] Schwartz C Turqueti-Neves A Hartmann S: Basophil-mediated protection against gastrointestinal helminths requires IgE-induced cytokine secretion. *Proc Natl Acad Sci U S A.* 2014;111(48):E5169–77. 10.1073/pnas.1412663111 25404305 PMC4260590

[14] Schwartz C Oeser K Prazeres da Costa C: T cell-derived IL-4/IL-13 protects mice against fatal *Schistosoma mansoni* infection independently of basophils. *J Immunol.* 2014;193(7):3590–9. 10.4049/jimmunol.1401155 25172500

[15] Sullivan BM Liang HE Bando JK: Genetic analysis of basophil function *in vivo*. *Nat Immunol.* 2011;12(6):527–35. 10.1038/ni.2036 21552267 PMC3271435

[16] Phythian-Adams AT Cook PC Lundie RJ: CD11c depletion severely disrupts Th2 induction and development *in vivo*. *J Exp Med.* 2010;207(10):2089–96. 10.1084/jem.20100734 20819926 PMC2947067

[17] Hammad H Plantinga M Deswarte K: Inflammatory dendritic cells--not basophils--are necessary and sufficient for induction of Th2 immunity to inhaled house dust mite allergen. *J Exp Med.* 2010;207(10):2097–111. 10.1084/jem.20101563 20819925 PMC2947072

[18] Otsuka A Nakajima S Kubo M: Basophils are required for the induction of Th2 immunity to haptens and peptide antigens. *Nat Commun.* 2013;4:1739. 10.1038/ncomms2740 23612279 PMC3644090

[19] Miyake K Shiozawa N Nagao T: Trogocytosis of peptide-MHC class II complexes from dendritic cells confers antigen-presenting ability on basophils. *Proc Natl Acad Sci U S A.* 2017;114(5):1111–6. 10.1073/pnas.1615973114 28096423 PMC5293035

[20] Tang H Cao W Kasturi SP: The T helper type 2 response to cysteine proteases requires dendritic cell-basophil cooperation via ROS-mediated signaling. *Nat Immunol.* 2010;11(7):608–17. 10.1038/ni.1883 20495560 PMC3145206

[21] Leyva-Castillo JM Hener P Michea P: Skin thymic stromal lymphopoietin initiates Th2 responses through an orchestrated immune cascade. *Nat Commun.* 2013;4:2847. 10.1038/ncomms3847 24284909

[22] Motomura Y Morita H Moro K: Basophil-derived interleukin-4 controls the function of natural helper cells, a member of ILC2s, in lung inflammation. *Immunity.* 2014;40(5):758–71. 10.1016/j.immuni.2014.04.013 24837103

[23] Huang FJ Ma YL Tang RY: Interleukin-4- and NACHT, LRR and PYD domains-containing protein 3-independent mechanisms of alum enhanced T helper type 2 responses on basophils. *Immunology.* 2016;149(2):238–51. 10.1111/imm.12636 27315109 PMC5011680

[24] Kim BS Wang K Siracusa MC: Basophils promote innate lymphoid cell responses in inflamed skin. *J Immunol.* 2014;193(7):3717–25. 10.4049/jimmunol.1401307 25156365 PMC4170007

[25] Siracusa MC Saenz SA Hill DA: TSLP promotes interleukin-3-independent basophil haematopoiesis and type 2 inflammation. *Nature.* 2011;477(7363):229–33. 10.1038/nature10329 21841801 PMC3263308

[26] Giacomin PR Siracusa MC Walsh KP: Thymic stromal lymphopoietin-dependent basophils promote Th2 cytokine responses following intestinal helminth infection. *J Immunol.* 2012;189(9):4371–8. 10.4049/jimmunol.1200691 23024277 PMC3478488

[27] Nakashima C Otsuka A Kitoh A: Basophils regulate the recruitment of eosinophils in a murine model of irritant contact dermatitis. *J Allergy Clin Immunol.* 2014;134(1):100–7. 10.1016/j.jaci.2014.02.026 24713170

[28] Cheng LE Sullivan BM Retana LE: IgE-activated basophils regulate eosinophil tissue entry by modulating endothelial function. *J Exp Med.* 2015;212(4):513–24. 10.1084/jem.20141671 25779634 PMC4387286

[29] Mukai K Matsuoka K Taya C: Basophils play a critical role in the development of IgE-mediated chronic allergic inflammation independently of T cells and mast cells. *Immunity.* 2005;23(2):191–202. 10.1016/j.immuni.2005.06.011 16111637

[30] Egawa M Mukai K Yoshikawa S: Inflammatory monocytes recruited to allergic skin acquire an anti-inflammatory M2 phenotype via basophil-derived interleukin-4. *Immunity.* 2013;38(3):570–80. 10.1016/j.immuni.2012.11.014 23434060

[31] Saeki K Satoh T Yokozeki H: α(1,3) Fucosyltransferases IV and VII are essential for the initial recruitment of basophils in chronic allergic inflammation. *J Invest Dermatol.* 2013;133(9):2161–9. 10.1038/jid.2013.160 23657464

[32] Iki M Tanaka K Deki H: Basophil tryptase mMCP-11 plays a crucial role in IgE-mediated, delayed-onset allergic inflammation in mice. *Blood.* 2016;128(25):2909–18. 10.1182/blood-2016-07-729392 27789480

[33] Meulenbroeks C van Weelden H Schwartz C: Basophil-derived amphiregulin is essential for UVB irradiation-induced immune suppression. *J Invest Dermatol.* 2015;135(1):222–8. 10.1038/jid.2014.329 25089660

[34] Ito Y Satoh T Takayama K: Basophil recruitment and activation in inflammatory skin diseases. *Allergy.* 2011;66(8):1107–13. 10.1111/j.1398-9995.2011.02570.x 21371044

[35] Wada T Ishiwata K Koseki H: Selective ablation of basophils in mice reveals their nonredundant role in acquired immunity against ticks. *J Clin Invest.* 2010;120(8):2867–75. 10.1172/JCI42680 20664169 PMC2912199

[36] Obata-Ninomiya K Ishiwata K Tsutsui H: The skin is an important bulwark of acquired immunity against intestinal helminths. *J Exp Med.* 2013;210(12):2583–95. 10.1084/jem.20130761 24166714 PMC3832932

[37] Reitz M Brunn ML Rodewald HR: Mucosal mast cells are indispensable for the timely termination of Strongyloides ratti infection. *Mucosal Immunol.* 2017;10(2):481–92. 10.1038/mi.2016.56 27381924

[38] Anyan WK Seki T Kumagai T: Basophil depletion downregulates Schistosoma *mansoni* egg-induced granuloma formation. *Parasitol Int.* 2013;62(6):508–13. 10.1016/j.parint.2013.07.003 23850838

[39] Noti M Kim BS Siracusa MC: Exposure to food allergens through inflamed skin promotes intestinal food allergy through the thymic stromal lymphopoietin-basophil axis. *J Allergy Clin Immunol.* 2014;133(5):1390–9, 1399.e1–6. 10.1016/j.jaci.2014.01.021 24560412 PMC4007098

[40] Hussain M Borcard L Walsh KP: Basophil-derived IL-4 promotes epicutaneous antigen sensitization concomitant with the development of food allergy. *J Allergy Clin Immunol.* 2017; pii: S0091-6749(17)30566-3. 10.1016/j.jaci.2017.02.035 28390860

[41] Muto T Fukuoka A Kabashima K: The role of basophils and proallergic cytokines, TSLP and IL-33, in cutaneously sensitized food allergy. *Int Immunol.* 2014;26(10):539–49. 10.1093/intimm/dxu058 24860117

[42] Noti M Wojno ED Kim BS: Thymic stromal lymphopoietin-elicited basophil responses promote eosinophilic esophagitis. *Nat Med.* 2013;19(8):1005–13. 10.1038/nm.3281 23872715 PMC3951204

[43] Venturelli N Lexmond WS Ohsaki A: Allergic skin sensitization promotes eosinophilic esophagitis through the IL-33-basophil axis in mice. *J Allergy Clin Immunol.* 2016;138(5):1367–1380.e5. 10.1016/j.jaci.2016.02.034 27233150

[44] Anthony RM Kobayashi T Wermeling F: Intravenous gammaglobulin suppresses inflammation through a novel T _H_2 pathway. *Nature.* 2011;475(7354):110–3. 10.1038/nature10134 21685887 PMC3694429

[45] Campbell IK Miescher S Branch DR: Therapeutic effect of IVIG on inflammatory arthritis in mice is dependent on the Fc portion and independent of sialylation or basophils. *J Immunol.* 2014;192(11):5031–8. 10.4049/jimmunol.1301611 24760152 PMC4025610

[46] Charles N Hardwick D Daugas E: Basophils and the T helper 2 environment can promote the development of lupus nephritis. *Nat Med.* 2010;16(6):701–7. 10.1038/nm.2159 20512127 PMC2909583

[47] Pan Q Gong L Xiao H: Basophil Activation-Dependent Autoantibody and Interleukin-17 Production Exacerbate Systemic Lupus Erythematosus. *Front Immunol.* 2017;8:348. 10.3389/fimmu.2017.00348 28396669 PMC5366357

[48] Yuk CM Park HJ Kwon B: Basophil-derived IL-6 regulates T _H_17 cell differentiation and CD4 T cell immunity. *Sci Rep.* 2017;7:41744. 10.1038/srep41744 28134325 PMC5278410

[49] Yazawa N Imaizumi T Makuuchi H: Treatment with Anti-FcεRIα (MAR-1) Antibody Prevents Acute Islet Allograft Rejection in a Murine Model. *Tokai J Exp Clin Med.* 2015;40(4):141–8. 26662664

[50] Schiechl G Hermann FJ Rodriguez Gomez M: Basophils Trigger Fibroblast Activation in Cardiac Allograft Fibrosis Development. *Am J Transplant.* 2016;16(9):2574–88. 10.1111/ajt.13764 26932231

[51] Rivellese F Suurmond J de Paulis A: IgE and IL-33-mediated triggering of human basophils inhibits TLR4-induced monocyte activation. *Eur J Immunol.* 2014;44(10):3045–55. 10.1002/eji.201444731 25070188

[52] Borriello F Longo M Spinelli R: IL-3 synergises with basophil-derived IL-4 and IL-13 to promote the alternative activation of human monocytes. *Eur J Immunol.* 2015;45(7):2042–51. 10.1002/eji.201445303 25824485 PMC4496336

[53] De Monte L Wörmann S Brunetto E: Basophil Recruitment into Tumor-Draining Lymph Nodes Correlates with Th2 Inflammation and Reduced Survival in Pancreatic Cancer Patients. *Cancer Res.* 2016;76(7):1792–803. 10.1158/0008-5472.CAN-15-1801-T 26873846

